# PD-1/PD-L1 checkpoint inhibitors in advanced hepatocellular carcinoma immunotherapy

**DOI:** 10.3389/fimmu.2022.1070961

**Published:** 2022-12-19

**Authors:** Qian Li, Jingjing Han, Yonglin Yang, Yu Chen

**Affiliations:** ^1^ Department of Anesthesiology, First Affiliated Hospital of Nanjing Medical University, Nanjing, China; ^2^ Department of Infectious Diseases, The Affiliated Taizhou People’s Hospital of Nanjing Medical University, Taizhou, China

**Keywords:** PD-1 inhibitor, PD-L1 inhibitor, hepatocellular carcinoma, immune checkpoint, tumor immune escape, immunotherapy

## Abstract

Hepatocellular carcinoma (HCC) has a high prevalence and mortality rate worldwide. Sorafenib monotherapy has been the standard of first-line treatment for advanced HCC for a long time, but there are still many shortcomings. In recent years, with the deepening of research on tumor immune microenvironment, researchers have begun to explore new approaches in immunotherapy, and the introduction of immune checkpoint inhibitors has brought fundamental changes to the treatment of HCC. Programmed cell death protein 1 (PD-1) is an immune checkpoint molecule that plays an important role in down-regulating immune system function and promoting tolerance. Programmed cell death ligand 1 (PDL-1) is involved in tumor immune evasion by binding to PD-1, resulting in failure of treatment. Currently, immunotherapy targeting the PD-1/PD-L1 axis has achieved unprecedented success in HCC, but it also faces great challenges, with its low remission rate still to be solved. For most patients with HCC, the PD-1/PD-L1 pathway is not the only rate limiting factor of antitumor immunity, and blocking only the PD-1/PD-L1 axis is not enough to stimulate an effective antitumor immune response; thus, combination therapy may be a better option. In this study, changes in the immune microenvironment of HCC patients were reviewed to clarify the feasibility of anti-PD-1/PD-L1 therapy, and a series of monotherapy and combination therapy clinical trials were summarized to verify the safety and efficacy of this newly developed treatment in patients with advanced HCC. Furthermore, we focused on hyperprogressive disease and drug resistance to gain a better understanding of PD-1/PD-L1 blockade as a promising treatment.

## 1 Background

Liver cancer is currently the fourth most likely cause of cancer death (1) Risk factors for HCC include hepatitis B virus (HBV) infection, hepatitis C virus (HCV) infection, alcohol consumption, smoking, obese nonalcoholic fatty liver, the consumption of aflatoxin-contaminated food, and type 2 diabetes (2).

HCC has a poor prognosis, with a 5-year survival rate of only 18% unless detected early in the disease.Early HCC can be cured by surgical resection, liver transplantation, or local interventional therapy; however, most patients are diagnosed with advanced disease and can only be treated primarily with systemic therapy (3). Multikinase inhibitors (MKI) targeting angiogenesis pathways have been first-line drugs for advanced treatment of HCC for a period of time (4). In recent years, with the continuous research on tumor immune microenvironment and the interaction between immune cells and tumor cells. The important role of the immune system in cancer progression has been recognized. At present, immunotherapy is becoming the fourth cancer treatment after surgery, chemotherapy, and radiotherapy.

PD-1 is a common immunosuppressive factor on the surface of T cells, PD-L1 is overexpressed on the surface of malignant tumor cells and binds to PD-1 to inhibit T cell proliferation and activation, making T cells inactivated and eventually inducing immune escape, resulting in treatment failure (5).PD-1/PD-L1-based pathways play an important role in cancer immunotherapy, and their inhibitors have made breakthroughs in treatment, bringing hope to HCC patients (6–8).

However, PD-1/PD-L1-inhibitors in the treatment of HCC still face major challenges. First, chronic HBV or HCV infection was once considered contraindicated by immunotherapy due to the possibility of virus reactivation (9). Second, targeted PD-1/PD-L1 immunotherapy has only been successful so far in a small number of patients, which is expensive and may be associated with immune-related adverse events (irAEs) (10).

The purpose of this study was to summarize the mechanism and application of PD-1/PDL-1 checkpoint inhibitors in HCC immunotherapy, and to reflect further on the side effects of treatment, in the hope of developing a promising combination therapy in the future, such that blocking the PD-1/PD-L1 pathway can play a more important role in the treatment of HCC.

## 2 Changes in the immune microenvironment of the liver induced by HCC

### 2.1 Immune microenvironment of the liver in normal state

As the main metabolic organ, the liver acts as an immune gatekeeper (11, 12), which is under the continuous attack of enteric-derived pathogens, metabolism-associated molecular patterns (MAMPs), Toll-like receptors (TLR), and various metabolites in normal biological processes. Thus, the liver has immunosuppressive polarity that weakens the T-cell-mediated antigenic response and is maintained by resident cell populations and peripheral leukocytes or bone marrow cells, including Kupffer cells, hematopoietic stem cells, hepatic stellate cells (HSC), dendritic cells (DC), regulatory T cells (Treg) and liver sinusoidal endothelial cells (LSECs) (13, 14).

Another mechanism contributing to natural liver immune tolerance is immune checkpoints. They include co-inhibitory molecules expressed by effector lymphocytes to prevent their overactivation, which can prevent T cells from being overactivated and lead to normal tissue damage and destruction, and play an important role in maintaining human immunologic tolerance (15).Another mechanism may include the establishment of a porous layer to isolate liver tissue from blood and limiting the ability to activate CD4^+^ T cells (16).

In conclusion, the liver can be protected from autoimmune injury through an innate immune tolerance or escape mechanism. Further, due to the uniqueness of the liver in promoting immunologic tolerance, it also promotes the growth of malignant tumor cells to some extent and prevents them from being recognized by the immune system (17).

### 2.2 Immune microenvironment of the liver under HCC

TME in HCC includes cancer cells, immune cell subsets, cytokine environment and extracellular matrix, which promote tumor progression (18). Under the influence of chronic inflammation, the continuous expression of different cytokines, the recruitment of immune cells to the liver, and the continuous enhancement of immunosuppression provide space for the growth of cancer cells, resulting in one or more of steps in cancer immunity cycle are damaged in the immunosuppressive TME and lead to inefficient tumor immune response, tumor resistance and disease progression (19).

HCC avoids the immune response through abnormal expression and impaired recognition of tumor antigens (20). At the same time, dysfunctional tumor-immune system interactions generate immunosuppressive tumor microenvironments (21), in which cell populations also play an important role in tumor immune escape through cell-cell contact, cytokine release and recognition, and other soluble factor interactions (13).

In the cancer immunity cycle of HCC, the direct killers of tumor cells are cytotoxic T cells and NK cells. Tumors can escape the immune surveillance of NK cells by immunoediting, altering the expression of ligands and produce NK cell resistant tumor variants (22). Also, studies have shown that in peripheral blood and intratumoural tissues of HCC patients, the number of tumour- infiltrating NK cells is decreased, and the function of NK cells in cytokine production and cytotoxicity is also impaired (23). This may be caused by the combination of some factors in TME with receptors on NK cells, such as TGFβ, adenosine and activin-A (24, 25). As for cytotoxic T cells, cancer cells can stimulate Tregs to inhibit CTL activity by releasing adenosine into TME and many other immunosuppressive mediators, including IDO, CXCL17, signal transducer, and transcriptional activator 3 (STAT3) to inhibit the activity of CTL and leading to poor production of IFN-γ (26–28). It can also induce CTL cell apoptosis by activating the PD1 receptor on the CTL surface, and hypoxia in TME can further induce the expression of PD-L1 in cancer cells, thereby increasing their resistance to CTL mediated lysis (29).

Also, immunosuppressive cells, such as tumor-associated macrophages (TAMs), myeloid-derived suppressor cells (MDSCs), and regulatory cells (Tregs), are key components of the TME that promotes HCC growth and invasion. These cells usually have both anticancer and procancer effects, and their interactions lead to immune escape of the tumor (30).

#### 2.2.1 TAMs

Macrophages are the main component of TME, some tumor promoting effects are attributed to these TAMs, and high levels of TAM are associated with poor prognosis in HCC patients (31, 32). The main roles of TAMs in the pathogenesis of HCC are (33):(1) Promoting the proliferation, invasion and metastasis of cancer cells in HCC;(2) promote angiogenesis in HCC;(3) promoting cancer cell stemness;(4)Inducing immunosuppression and weaken therapeutic effect

The TAM subgroup of HCC consists of two groups, namely those that undergo classical activation (M1) and those that undergo alternative activation (M2). M1 produce anti-tumor factors and induce anti-tumor immune response while M2 produce tumor growth factors and produce angiogenic molecules, resulting in tumor promotion. The dynamic changes of liver macrophage phenotype are related to the occurrence and progression of cancer. And in HCC, under the influence of various factors, for example, Nogo-B (34) was suggested to promoting TAM M2 polarization by enhancing Yap/Taz pathway, the TAM subgroup in HCC is mainly M2 subtype (35), so as to further promote the tumor promoting effect of TAMs.

#### 2.2.2 MDSCs

In HCC, The immunosuppressive activity of MDSC in tumor microenvironment mainly includes (30) (a) inducing Tregs’ differentiation and expansion; (b) depriving of essential amino acids from T cells and affecting their function, survival, and trafficking; (c) inducing oxidative stress to mediates cancer progression; (d) expanding signal transduction of immune checkpoint and reducing NK cell cytotoxicity. MDSC can induce T cell apoptosis by expressing galactose lectin-9 and binding to TIM-3 on T cells. MDSCs can also produce IL-10 to inhibit TLR-ligand-induced IL-12, which leading to the inhibition of T-cell stimulating activity of DCs in HCC (36).

#### 2.2.3 Tregs

Another well-known example is Tregs cells, whose density in the liver is associated with poor prognosis and metastatic disease tendency of HCC (37, 38). First, through their high high-affinity IL-2 receptor α chain (CD25),tregs consumes IL-2 in TME to inhibit the proliferation and activation of T cells; secondly, Tregs that express CTLA-4 are more likely to bind to costimulatory molecules (CD80 and CD86) on APC, thus depriving T cells of the stimulation signal. In addition, Tregs secretes inhibitory cytokines such as TGF- β to weaken the anti-tumor effect mediated by CD4+T cells, CD8+T cells and NK cells. What’s more, Tregs directly kill responder T cells or APCs through producing granzyme and perforin (39).

Antitumor immune responses can also be inhibited by upregulating the expression of checkpoint inhibitors, such as CTLA-4, Tim-3 and its ligand, the adenosine A2a receptor, PD-1 and PD-L1 (19, 40–42). Also, vascular endothelial growth factor (VEGF) produced by cancer cells or MDSCs can lead to abnormal formation of the tumor vascular system, and indoleamine2,3-dioxygenase (IDO) reduce the effects of the effects of immunoglobulin-mediated opsonin effects (43, 44).

In conclusion, various mechanisms interact to form the immunosuppressive microenvironment of HCC. Among them, the role of the PD-1/PD-L1 pathway cannot be ignored, and it has become one of the most important therapeutic targets for preventing tumor progression.

## 3 Role of the PD-1/PD-L1 pathway in the tumor microenvironment provides ideas for immunotherapy

PD-1 is an immune checkpoint molecule, belonging to the CD28 family, whose role is to reduce T cell activity during the immune response and prevent autoimmune injury by binding to its ligand PD-L1 or PD-L2 to prevent the stimulation signal of the T cell receptor (TCR) (45). PD-1 is expressed on a variety of immune cells, such as activated T cells, B cells, NK cells, and dendritic cells. PD-L1.PDL-1 is expressed in tumor cells and antigen presenting cells APCs while PD-L2 is mainly expressed on dendritic cells and macrophages. In the case of chronic infection, prolonged antigen exposure leads to permanent expression of PD-1, thus limiting immune-mediated pathogen clearance (46).

PD-1 contains two structural motifs in its cytoplasmic tail: immunoreceptor tyrosine-based inhibitory motif (ITIM) and immunoreceptor tyrosine-based Switch motif (ITSM). When PD-1 binds to its ligand, ITSM is phosphorylated to activate intracellular pathways to exert immunosuppressive activity. However, the PD-1/PD-L1 axis inhibition mechanism is different between T cells and B cells.

**Figure 1:**
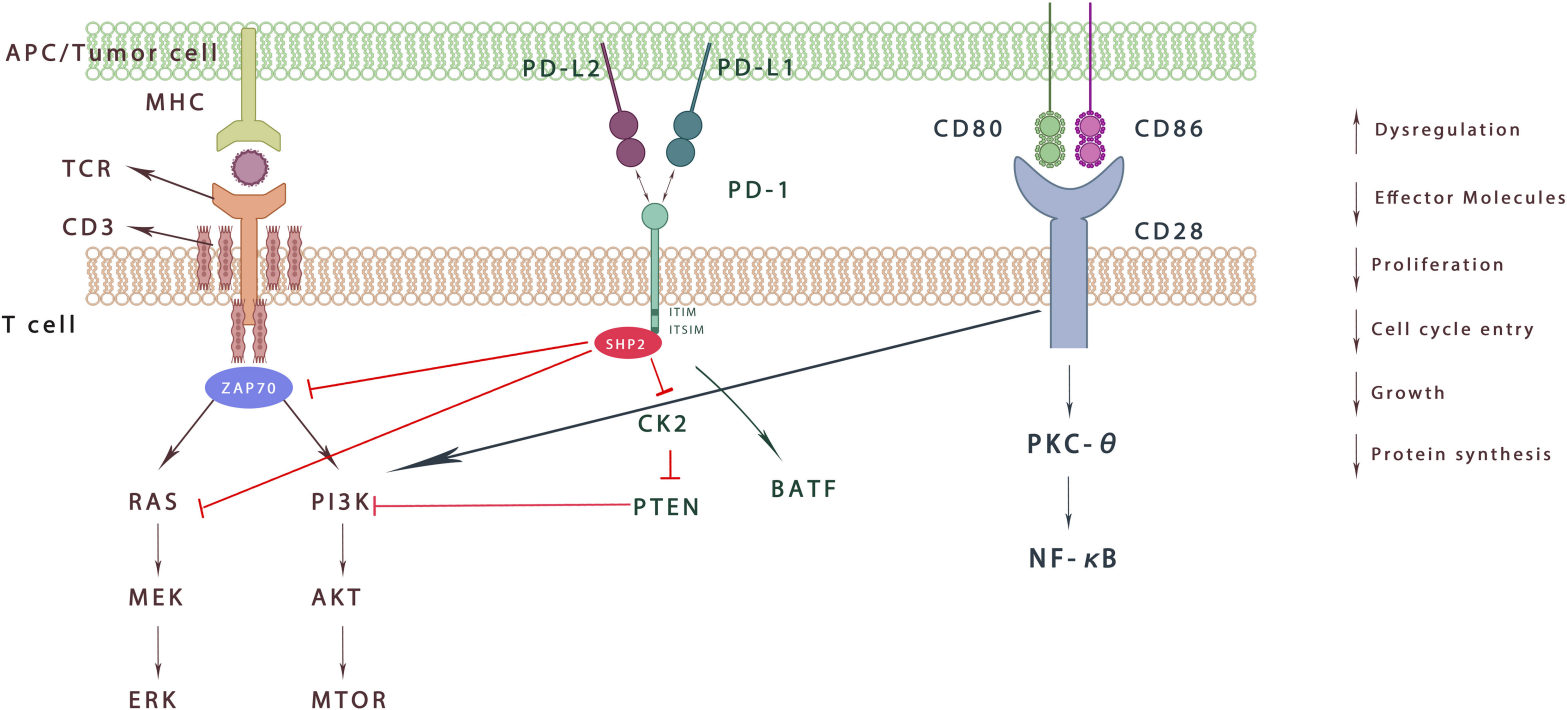
Effect of PD-1 signaling on T cells. When binding to PD-L1 and PD-L2, ITSM is phosphorylated and recruits SHP-2, which inhibits downstream signaling through dephosphorylated kinases. It further weakens ZAP70 phosphorylation and decreases the RAS-MEK-ERK/PI3K Akt mTOR pathway. In addition, PD-1 activation increases the expression of BATF. Overall, PD-1 signaling aggravates the imbalance of immune microenvironment, inhibits T cell proliferation, decreases effector molecules, and protein synthesis.

In T cells, the phosphorylated ITSM recruits SHP-1/2 molecules into the C-terminal ITSM, but only SHP-2 interacts with PD-1 during T cell activation to produce real-time effects (47). It further antagonizes the positive signals generated through TCR and CD28 and affects downstream signalling pathways such as PI3K–AKT, RAS, and ERK (48). PD-1 can also inhibit T cell function by increasing the expression of transcription factors such as basic Leucine zipper transcription factor (ATF-like, BATF) which further antagonizes effector transcriptional programs (**Figure 1**). Furthermore, PD-1 signaling regulates T cell function by preventing glycolysis and promoting lipid degradation and β-oxidation (16). The above actions ultimately affect the activation, longevity and proliferation of T cells, leading to the decrease in tumor necrosis factor (TNF), Interferon-γ(IFN-γ), interleukin (IL)-2 and other cytokines, as well as metabolic changes, providing a way for cancer cells to escape the immune response (49). PD-1/PD-L1 expression also exists in Tregs, another T cell subtype, aggravating suppression and exhaustion of immune status in TME (16). It has been reported that PD-L1 induces the differentiation, maintenance and function of iTregs by maintaining and increasing the expression of Foxp3. Also, PD-L1 can transform original CD4+T cells into Tregs by downregulating Akt, mTOR and ERK2 and simultaneously up regulating PTEN (50, 51).What’s more, endothelial cells can enhance T reg function through PD-1/PD-L1 axis, and Tregs can also inhibit autoreactive B cells through PD-1/PD-L1 axis (51).

In B cells, after PD-1 activation, SHP-2 is recruited to the C-terminal of PD-1 to dephosphorylate BCR pathway molecules, including Igα/β and SγK, which inhibits PI3K, ERK and PLC γ 2 pathways, leading to Ca2+ disorder and stagnation of B cell growth (39). It is reported that B cells overexpressing PD1 can induce T cell dysfunction through an IL10 dependent pathway, thereby creating conditions conducive to tumor progress (52). In addition, *in vitro*, PD-1+B cells can inhibit the expansion of T cells and reduce their viability, while PD-L1 blockade can increase the proliferation and viability of T cells. It is worth noting that, these PD-1+B cells do not express high levels of IL – 10 (53). Other experiment has shown that IgA+ plasma cells that highly express PD-L1 and IL-10 accumulate in people and mice with nonalcoholic steatohepatitis, directly inhibiting CTL activation and finally promoting hepatocellular carcinoma. IgA+ B cells continuously overexpress PD-L1, producing large amounts of IL-10 and TGF- β and has immunosuppressive effect on the proliferation and function of CD8+T cells. This suggests that PD-L1+B cells may be a subset of Bregs and play a powerful immunosuppressive function against T cell reaction (54).

What’s more, recent study has shown that human TAM also expresses PD-1, which increases with the increase of disease stage and show a surface profile similar to M2 (55). The expression of PD-1 on PD-1+TAM is associated with decreased phagocytosis of macrophages and increased invasion of cancer cells, which may lead to poor prognosis of cancer (56). In addition, it has been reported that PD-L1 is preferentially expressed on macrophages rather than cancer cells (56), and in the mice model, TAMs can reduce the anti-cancer immune response of CD8^+^ and CD4^+^ T cells by expressing PD-L1 (57). Although the importance of PD-L1/PD1 expressed in TAM is still not fully understood, targeting macrophages expressing PD-L1 in HCC can be used as a strategy to improve the effect of immunotherapy.

Moreover, the specific microenvironment generated by tumor releasing factors and hypoxia conditions can induce the expression of PD-L1 in MDSCs (58).They can inhibit the activation of T cells by binding with PD-1 on T cells and are immune suppressive toward T cells activated by anti-CD3 and anti-CD28 *in vitro*. Also, MDSC can activate PI3K/AKT/NF-κB pathway in B cells through PD-1/PD-L1 axis to induce a group of PD-1 − PD-L1+Bregs exerting immunosuppressive effects (59). It is also reported that the expression of PD-1 on MDSC can be induced by LPS, which further promote tumor development and recurrence through regulating its proliferation and inhibition molecules (60).In conclusion, the inhibition of PD-1/PD-L1 on MDSCs may be crucial for treatment in HCC.

**Figure 2:**
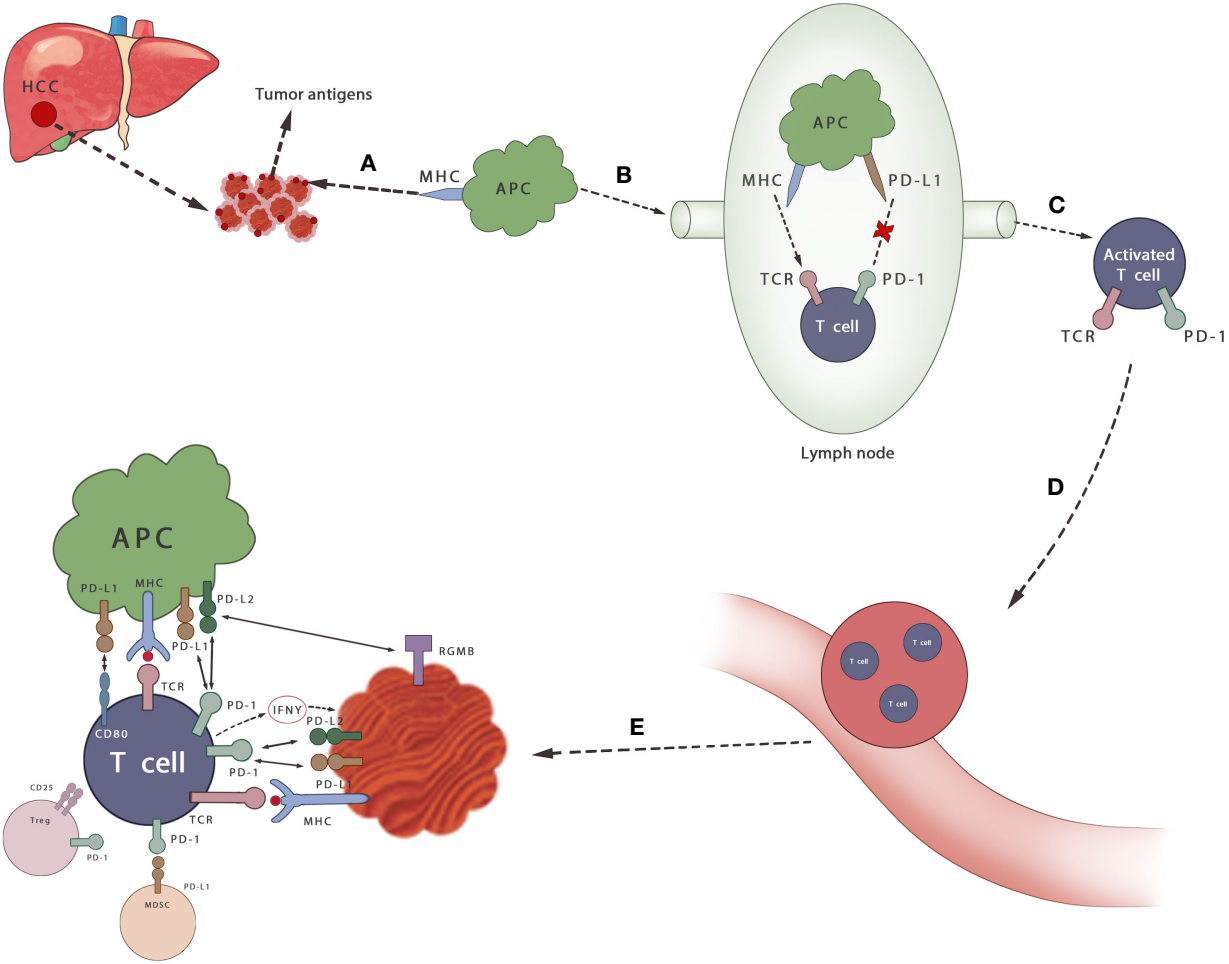
Mechanisms of PD-1/PD-L1 axis in HCC. **(A)** Cancer antigen is released from HCC cells and recognized by APCs **(B)** APCs that capture antigens migrate to lymph nodes **(C)** APC presents antigen to immature T cells through TCR and activates T cells, which can be inhibited by PD-1/PD-L1 axis **(D, E)** T cells infiltrate to the tumor. Activated T cells secrete IFN γ and stimulate cancer cells to express PD-L1 and protect themselves. By binding its ligand PD-L1 or PD-L2, it can prevent the stimulation signal of TCR, reduce the activity of T cells and prevent autoimmune damage during the immune response. MDSCS inhibits the activation of T cells by binding to PD-1 on T cells. Tregs also inhibits the proliferation and activation of T cells.

In general, PD-1/PD-L1 axis plays a huge role in the immunosuppressive microenvironment of HCC (**Figure 2**). It is believed that PD-1/PDL-1 inhibitors can increase, the sensitivity of their mediated killing effect by removing the immunosuppression of antitumor T cells. Furthermore, T cells can proliferate and infiltrate into the TME to induce the antitumor response (61). It can also promote the activation of tumor-draining lymph nodes (TDLN) CD8^+^ T cells and restore the vitality of tumor-resident predysfunctional CD8^+^ T cells (62). The activity of the mTOR pathway is also enhanced after anti-PD-L1 antibody treatment, and transcriptome maps show that macrophages are activated by multiple pathways, becoming activated, inflammatory, proliferative, and long-lived macrophages, which may be another anticancer approach (63, 64).

## 4 Clinical evidence supporting PD-1/PD-L1 blockade for the treatment of HCC

Despite initial concerns that ICIs could cause viral outbreaks in HCV or HCC associated with HBV, the first pivotal trials clearly confirmed the safety of ICIs in these patients. The researchers tested the antiviral activity of CTLA-4 blocker in HCV infected patients for the first time, and found that there was no fulminant hepatitis in the patients, proving the good security of ICIs (65). In Checkmate-040 trial, the results of the phase 1/2 dose escalation and expansion cohort shows that no patient with HBV infection has hepatitis attack, and this is the first time that chronic HBV patients have been included in the clinical trial of immune checkpoint blockers, which paves the way for subsequent immunotherapy efficacy tests in HCC (66).

### 4.1 Nivolumab

The first anti-PD-1 drug to be used for HCC was nivolumab, a fully human IgG4 monoclonal antibody that blocks PD-1 interactions with PD-L1 and PD-L2. In CheckMate 040, a multicenter global phase III clinical trial, nivolumab showed good efficacy and safety among patients with advanced liver cancer who received or did not receive sorafenib in advance. Furthermore, there was no virus outbreak in patients with HCV or HBV hepatitis (67, 68). Based on the CheckMate 040 trial results, the FDA approved nivolumab as second-line systemic therapy for patients pretreated with sorafenib. Later, in order to compare the efficacy of nivolumab versus sorafenib as first-line treatment in patients with advanced HCC, investigators initiated the Checkmate 459, a phase III randomized multicenter study. The results showed that compared to sorafenib, the use of nivolumab as a single first-line therapy did not improve overall survival, but had a good clinical efficacy and safety profile and a higher complete alleviation response rate was observed, which improved the patients’ quality of life (69). Based on these results, the indication for second-line treatment of liver cancer with nivolumab was withdrawn and the FDA rejected continued accelerated approval of nivolumab by a vote of 5:4. Therefore, for people who are inhibited from using tyrosine kinase inhibitor and antiangiogenesis drugs or patients at significant risk, nivolumab can be considered as a treatment option. Currently, nivolumab is being evaluated in several other ongoing trials: in checkmate-9dx, monotherapy is used as an adjuvant therapy for liver cancer (NCT03383458) and in combination with ipilimumab in the treatment of patients treated with HCC (NCT01658878).

### 4.2 Pembrolizumab

Another PD-1 inhibitor, pembrolizumab, was shown to be effective and tolerable in patients with advanced HCC who had previously been treated with sorafenib in cohort 1 of The Keynote-224 clinical trial, and was subsequently approved by the FDA (70). However, in Keynote-240, a phase 3 randomized, double-blind randomized, double-blind clinical trial to evaluate the efficacy and safety of pembrolizumab in patients with advanced HCC who have failed sorafenib treatment, Overall Survival(OS) and Progression-Free Survival (PFS) were not statistically significant, while some clinical benefits were shown (71, 72).Due to the failure of Keynote-240, NCCN changed the evidence category of pembrolizumab for second-line treatment of HCC from 2a to 2b in the second edition of the guidelines for hepatobiliary tumors in 2019 (73). In Keynote-394, pembrolizumab became the first and currently the only phase III trial in the world to achieve positive results with single use of the PD-1 inhibitor for advanced HCC. This is a randomized, double-blind, Phase III trial that enrolled patients with HCC who were intolerant or advanced after sorafenib or oxaliplatin therapy. The protocol was pembrolizumab (200 mg every three weeks intravenously for up to 35 cycles) +BSC (optimal supportive treatment) or placebo (every three weeks intravenously) +BSC were randomized 2:1. Compared to the control group, the median OS and PFS were significantly longer and the risk of death was reduced by 21%. The 2-year OS rate of the pembrolizumab group was higher than that of the control group (24.9%). Primary endpoint OS, secondary endpoint PFS, and objective response rate (ORR) were all positive (74). This provides a new option for the treatment of HCC.

### 4.3 Camrelizumab

Camrelizumab (SHR-1210) is also a human IgG4 monoclonal antibody against PD-1. In a multicenter, open, randomized phase 2 trial in China, 217 patients who received camrelizumab had an ORR of 14.7%, median PFS of 2.1 months, median overall survival (mOS) of 13.8 months and an overall survival rate of 74.4% at 6 months. The most common reatment-related adverse events were increased aspartate aminotransferase (5%) and reduced neutrophil count (3%) (75).

### 4.4 Atezolizumab

Atezolizumab is a monoclonal antibody against PD−L1. According to the phase III imbrave150 study, atezolizumab combined with bevacizumab reduced the risk of death (OS) by 56% and the risk of disease deterioration or death (PFS) by 40% compared to sorafenib. Moreover, atezolizumab and bevacizumab are well tolerated and toxicity is easy to control (76).The combination of atezolizumab and bevacizumab has been approved by the US FDA for clinical application in May 2020, becoming the first immunotherapy scheme approved for first-line treatment of unresectable HCC (77). In the phase III clinical trial COSMIC-312, which was designed to compare atezolizumab + cabozantinib and sorafenib monotherapy, its main endpoints, namely, significant improvement in PFS, were achieved. However, the other main endpoint, OS, was not achieved in the combination treatment group. Therefore, this regimen is considered to be a treatment option for some advanced patients with HCC, and more studies are needed to prove it in the future (78).

### 4.5 Durvalumab

Durvalumab, a humanized IgG1 monoclonal antibody against PD-L1, was evaluated as monotherapy in non-resectable HCC in a Phase I/II clinical trial (79) (**Table 1**). Recently, a Himalaya phase III study evaluated the effect of first-line treatment of liver cancer patients with durvalumab + tremelimumab (D + T), durvalumab monotherapy or sorafenib. The results showed that compared to sorafenib, both D + T combination therapy and durvalumab monotherapy significantly improved the survival of HCC patients (80). The first edition of the NCCN guidelines for liver cancer in 2022 has recommended durvalumab (class 2A evidence) for the first-line treatment of advanced liver cancer.

**Table 1:** Clinical trial results of anti PD1/PD-L1 monotherapy for HCC.

Drug	Clinical trial database number	Phase	ORR%	DCR %	mDOR, months	mOS, months	mPFS, months
Nivolumab	NCT01658878	I/II	15/20	58/64	17/9.9	15.0/NR	-/4.0
Nivolumab	NCT02576509	III	15.4	55	23.3	16.4	3.7
Pembrolizumab	NCT02702414	II	18.3	61.5	21	13.2	4.9
Pembrolizumab	NCT02702414	II	15.7	56.9	16.2	16.9	4.3
Pembrolizumab	NCT02702401	III	18.3	62.2	13.8	13.9	3.0
Camrelizumab	NCT02989922	II	14.7	44.2	NR	13.8	2.1
Durvalumab	NCT01693562	I/II	10.3	33.3	–	13.2	–
Durvalumab	NCT03298451	III	17.0	54.8	16.8	16.6	3.7

ORR, objective response rate; DCR, disease control rate; mDOR, median duration of response; mOS, median overall survival; mPFS, median progression-free survival; NR, Not Reached.

In addition to clinical efficacy, PD-1 can also be used as a prognostic marker for HCC to some extent. More than 10 years ago, researchers found that among surgically resected HCC patients, DFS and OS of patients with positive PD-L1 expression were significantly lower than those with negative PD-L1 expression (81), which has been repeatedly confirmed in recent years. All of this provides a new approach for the treatment of HCC.

## 5 Challenges in anti-PD1/PD-L1 therapy

### 5.1 Drug resistance

However, although anti-PD-1/PD-L1 therapy has achieved a strong antitumor effect in some patients, it has been successful only in a small number of patients, and most patients cannot benefit from a-PD-1/PD-L1 therapy (82, 83). In addition, many respondents will have acquired resistance after the initial response (84). Therefore, it is necessary to understand the mechanism of drug resistance to improve the efficacy of anti PD1/PDL1 therapy. For non-responders, the PD-1 signaling is not a rate-limiting rheostat of the tumor immune cycle, and it is not enough to restore antitumor immunity by blocking PD-1 or PD-L1, which is expensive and can be accompanied by immune-related adverse events (70) (irAEs) (**Table 2**).

**Table 2:** Immune-related adverse events related to the immune system caused by anti-PD-1/PD-L1 therapy.

System	Syndromes
Integumentary system	Rash, itchy skin, multiforme erythema
Neuromuscular system	Myasthenia gravis, necrotizing myopathy, vascular neuropathy, multiple neuropathy
Digestive system	Diarrhea, abdominal pain and occasional fever, colitis, hepatitis
Endocrine system	Hypophysitis, abnormal thyroid function, adrenal insufficiency
Circulatory system	Atrioventricular block, myocarditis, cardiomyopathy, myocardial fibrosis, heart failure, and pericardial disease.
Hematological system	Anemia, neutropenia, lymphopenia

**Figure 3:**
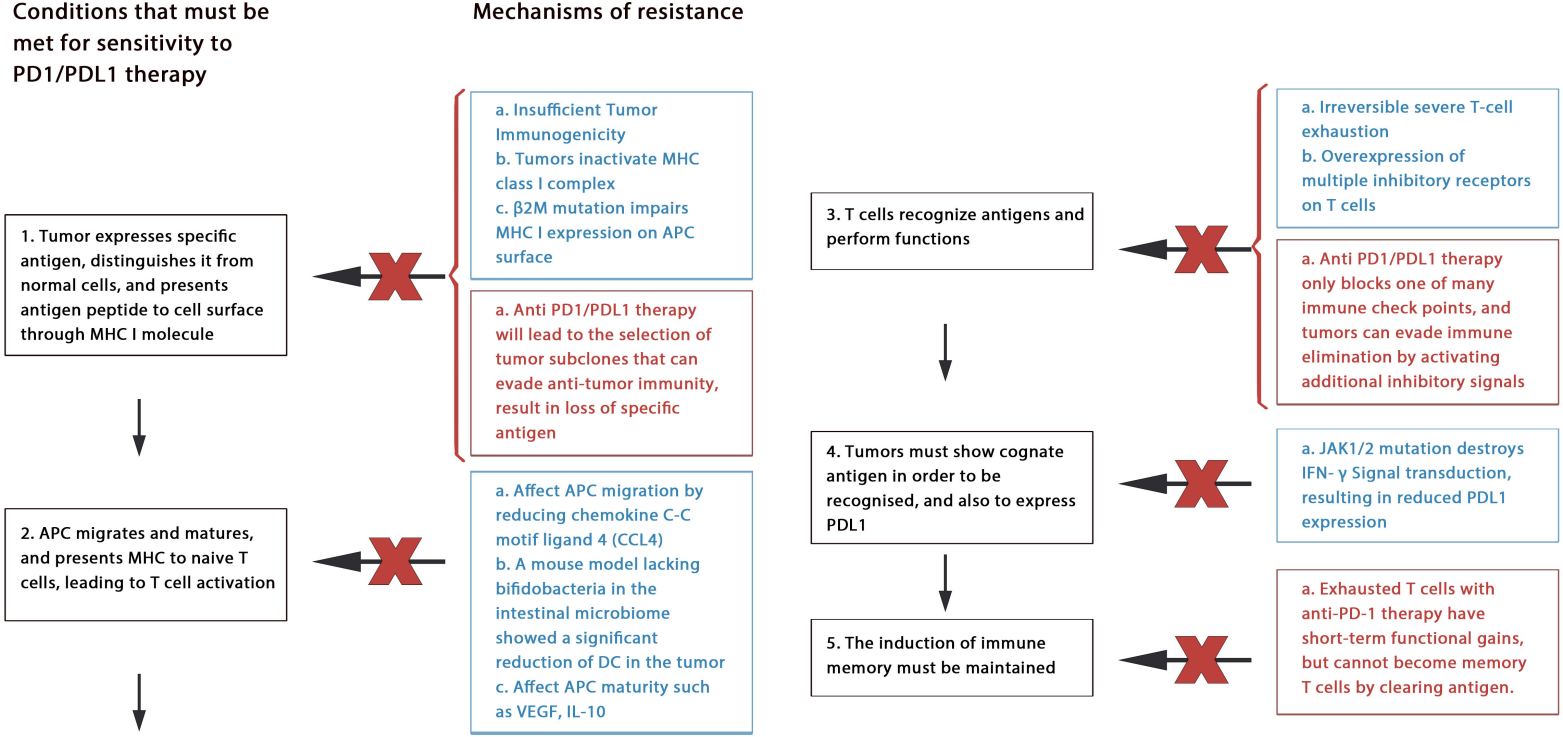
Mechanism of drug resistance: Blue text represents primary resistance, red text represents acquired resistance.

Many preclinical and clinical studies have shown that anti-PD-1 therapy often leads to congenital and acquired drug resistance (85), leading to tumor recurrence and treatment failure in patients with patients with HCC (**Figure 3**).

#### 5.1.1 Primary resistance

Anti-PD-1/PDL-l therapy plays an antitumor role by enhancing the function of existing CD8^+^ T cells. However, tumor rejection can be avoided by many ways to block the antitumor effect of T cells. This may include tumor immunogenicity deficiency, MHC dysfunction, irreversible T cell depletion, creating an immunosuppressive microenvironment (5, 86).

Through deep sequencing of human cancer mutations, researchers realized that tumor-specific neoantigens generated by nonsynonymous mutations play an important role in inducing tumor-specific immunity (86, 87). As the efficacy of anti-PD-1 therapy depends on whether there are tumor antigen-specific T cells in tumor tissue, tumors need to express specific antigens to distinguish themselves from other untransformed parts. If this fails, low or lack of antitumor immunity can lead to ineffective anti-PD-1 treatment. This means that tumors with poor immunogenicity may have great resistance to anti-PD1 treatment, as has been proven in recent studies (88). Furthermore, protein molecules such as Beta2 microglobulin (β2M), large multifunctional protease (LMP) are important components of tumor antigen processing and/or presentation, and their genetic changes will also cause resistance. For example, β2M participates in folding and transporting MHC I molecules, their mutation can alter the function of MHC I, which finally leads to the resistance (89).

Another factor is irreversible T cell failure. Long-term exposure to antigen stimulation can induce T cell exhaustion, and exhausted CD8^+^ T cells express multiple inhibitory receptors. It is speculated that the anti-PD-1/PDL-1 antibody can only overcome a part of the inhibitory signals, but the inhibitory axis mainly acts and hinders T cell function of T cells (90).

Other mechanisms may include classic oncogene mutation (such as EGFR mutation, KRAS mutation, PTEN deletion, or mutation); intestinal flora disorder (91); resistance of IFN-γ signaling; and limited memory T-cell induction (90, 92).

#### 5.1.2 Acquired resistance

Despite achieving initial results, some patients have developed drug resistance or relapse after blocking treatment with PD-1/PDL-1, which is called acquired resistance, occurs mainly through cancer immunoediting.

Neoantigens produced by non-synonymous mutations are vital for inducing T cell responses, causing tumor cells to eliminate their subsets with high immunogenicity of mutant-related antigens (93). Additionally, tumor cells can selectively eliminate antigen subsets with high immunogenicity through reduced gene expression or deletion of mutant allele deletion (94).

In addition, epigenetic changes in tumor cells are also associated with tumor immune escape. For example, malignant cells evade recognition by the immune system by selectively reducing or silencing the expression of tumor associated antigen (TAA) and costimulatory molecules (95). Within tumors epigenetic changes, the methylation of CpG islands in the promoter region of tumor suppressor genes is very common, histone acetylation, ADP ribosylation, ubiquitination can also be seen, resulting in the resistance together (96).

Furthermore, exhausted T cells with anti-PD-1 therapy have short-term functional gains, but cannot become memory T cells by clearing antigen. This may also lead to acquired resistance (97).

Currently, treatment strategies for PD-1 resistance mainly focus on enhancing T cell initiation, reversing T cell failure, increasing T cell infiltration, and improving the immunosuppressive microenvironment (90). Scholars believe that unless combined with other strategies, tumors can suddenly appear at any defenseless step of the cancer immune cycle during anti-PD1/PDL1 therapy (98).

### 5.2 Hyperprogressive disease

In 2017, Champiat defined hyperprogressive disease (HPD) as the at least twice increase in tumor growth kinetics (TGK) that occurred before or after targeting PD−1/PDL1 immunotherapy (99). After that, it mainly to the rapid and aggressive tumor progression pattern when patients begin immunotherapy (100), which is closely associated with poor prognosis (101). However, its mechanism has not been fully elucidated at present. Evidence shows that the incidence of HPD caused by PD-1/PD-L1 blockade single using is 4%–29% (102), and HPD has been reported in the treatment of HCC as well (103–105).

At present, it is believed that the abnormal occurrence of HPD after the use of PD-1/PD-L1 blockade may be caused by redundancy of immune signaling pathways and the pleiotropic effects of the factors involved in immunity (100). Its physiological mechanism may include (1): regulatory T cell proliferation leading to an immunosuppressive TME (106); (2) inhibition of the PD-1/PD-L1 axis leading to up-regulation of immunosuppressive checkpoints in antitumor CD8^+^ T cells, inhibiting immune response to cancer cells (107, 108); (3) increasing the number of Tsens (4); induction of immunologic tolerance *via* Fc receptors, such as FcγR I, by regulating inflammatory cytokines and production of M2 macrophages, promoting tumor growth; (5) triggering of uncontrolled inflammation mediated by T helper cell 1 (TH1) and TH17, which is conducive to cancer immune escape and accelerate tumor growth; (6) amplification of MDM2/4 and EGFR mutation; (7) altering the balance of immunosuppressive cytokines; (8) increasing of innate lymphoid cells 3 (ILC3); and (9) altering the specificity and affinity of ICIs due to the high level of LDH and the acidic environment, which can affect the function of antibodies and the conformation of antigens.

Patients with HPD generally have a worse prognosis, so it is necessary to pay closer attention to the patient’s immediate condition and evaluate the therapeutic effect of ICIs. The existing cancer disease monitoring and evaluation system urgently needs to be changed as it does not include TGK, which can be used as early marker of HPD (109). Currently known HPD biomarkers are mainly biomarkers of the tumor cell (such as MDM2/4 oncogene, EGFR), of the TME (Treg cells, M2 TAMs cells), and laboratory biomarkers (neutrophils-to-lymphocyte ratio, NLR) and clinical indicators (110). However, the value of these biomarkers in predicting HPD has not been validated. Early identification of HPD and replacement of ICI in time may be the only remedy to avoid the patient from the risk of getting HPD. Furthermore, it has been proposed that the baseline immune profile and the analysis of the tumor growth dynamics on treatment can guide optimal selection and identify the rapid tumor growth induced by PD-1 inhibitors in HCC patients earlier (104).

Moreover, some researchers have pointed out that combining NK cells with ICIs may be an option for patients with HPD. Furthermore, chemotherapy may help prevent resistance to immune checkpoint inhibitors (ICI) and HPD in patients, a possibility worthy of further investigation (109).Therefore, combination therapies may be a good choice.

## 6 Combined application of PD-1/PD-L1 blockade

### 6.1 Combination with tyrosine kinase inhibitors

As the cornerstone of cancer treatment, tyrosine kinase inhibitors (TKIs) have without a doubt opened an era of systemic treatment of HCC, and their combined treatment with anti PD1/PD-L1 agents has attracted much attention.

The occurrence of HCC is driven by abnormal activation of different intracellular pathways, which involves the role of tyrosine kinase (TK) protein receptors and non-TK receptors (110). TKIs inhibit tumor neovascularization and tumor growth by inhibiting key signaling pathways in the pathogenesis of HCC. For example, it can inhibit a variety of cell surface tyrosine kinases in the MAPK cascade, such as VEGFR, platelet derived growth factor receptor- β (PDGFR)-β, and downstream intracellular serine/threonine kinases, which are involved in signal transduction, proliferation, angiogenesis and apoptosis of tumor cells (110).

In addition, TKIs may also have immunomodulatory effects. For example, sorafenib (4) can enhance the activity of tumor specific effector T cells and reduce the inhibitory immune cell population. regorafenib can inhibit the infiltration of TAM by inhibiting the TIE2 pathway, induce sustained M1 polarization and reverse M2 polarization, and also induce CD8+T cells to activate and inhibit Tregs (111). In addition, it can also reduce immune escape by inhibiting the expression of PD-L1 on tumor cells (112). Therefore, the immunomodulatory effect of TKI can enhance the efficacy of anti PD-1/PD-L1 therapy. Furthermore, combined therapy can be applied to some patients with non-resectable HCC, to achieve resectable lesions (113).

In the Phase Ib study KEYNOTE-524, the combination of lenvatinib and pembrolizumab achieved an ORR of unresectable HCC of 36.0% (114), but its Phase III trial LEAP-002 did not reach the primary end point of the study, but showed a trend toward beneficial activity.

Another combination, camriezumab combined with alpatinib, has shown significant efficacy in the treatment of advanced liver cancer in the RESCUE experiment (115). Following the previous success, the international multicenter Phase III clinical trial (SR-1210-iii-310)aimed at comparing camrelizumab combined with apatinib vs sorafenib as first-line therapy for advanced HCC has been carried out, and recently it was announced that the primary endpoint was reached. The primary endpoint was PFS and OS, and the secondary endpoint was ORR, DCR, DOR and safety. Compared with the sorafenib (**Table 3**), the combination treatment group can significantly reduce the risk of disease progression or death, and generally well tolerated (116). Most adverse reactions occurred are not serious events and controllable. This is the first time in the world to use PD-1 monoclonal antibody combined with small molecule EGFR-TKI for first-line treatment in patients with advanced hepatocellular carcinoma, which achieved complete success. Furthermore, phase III clinical research on the combination has been carried out in the field of combing with TACE and other treatments, and the research results are also worth looking forward to.

**Table 3:** Camrelizumab combined with apatinibhad vs sorafenib.

	Camriezumab combined with alpatinib	Sorafenib
**OS, months**	22.1	15.2
**PFS,months**	5.6	3.7
**ORR %**	25.4%	5.9%
**DCR %**	78.3%	53.9%
**≥3 TRAE %**	80.9%	52.4%

EGFR TKIs improve the survival rate of HCC patients, but the rapid genetic mutations and epigenetic mutation of EGFR tyrosine kinase domain and other drug resistance mechanisms that do not depend on EGFR make many patients develop drug resistance in long-term treatment. For drug resistant patients, accurate management should be taken on the basis of clear drug resistance mechanism to maximize the survival benefits of patients. At present, new detection technologies such as liquid biopsy are gradually mature, which can monitor the occurrence of drug resistance and diagnose the mechanism of drug resistance, and help to better guide the treatment of drug resistance. In addition, the combination of drugs mentioned above also brings new hope to these patients. However, combination therapy also faces some obstacles, for example, excessive inhibition of tumor blood vessels may lead to tumor invasion and metastasis by inducing tumor to transform into hypoxia resistant phenotype (117). Therefore, future research should investigate how to obtain accurate treatment, identify specific and extremely sensitive biomarkers, and select the most suitable combined treatment for individuals.

### 6.2 Combination with antiangiogenic agents

In TME, the interaction between tumor blood vessels and protumoral immune cells seriously interferes with anticancer immunity, promotes tumor progression and impairs the efficacy of ICIs. Abnormal tumor neovascularization not only generates endothelial barrier, prevents T cells from infiltrating into the tumor, damages the function of T cell effector, causes T cell apoptosis, but also promotes the escape of protumoral immune cells, thereby promoting tumor angiogenesis (118). In addition, the tumor vascular system inhibits and kills CTL by expressing various immunosuppressive molecules, such as PD-L1 and Fas ligand (119). VEGF is a key driver of tumor angiogenesis. It enhances the mobilization and proliferation of a variety of cells, including the release of regulatory T cells and immunosuppressive cytokines to reduce the proliferation and function of CD8+ cells (119). In addition, VEGF can also inhibit the differentiation, maturation and antigen presentation of DCs, and increase T cell depletion and reduce the proliferation of cytokines produced by CTL through up regulating transcription factor TOX (120).

Some clinical studies show that there is resistance to immunotherapy in tumors with high VEGF (118, 121), and HCC is a highly vascular tumor. The interaction between angiogenesis and tumor immunity indicates that remodeling tumor vascular can improve the efficacy of anti PD-1 immunotherapy. Anti VEGFR2 therapy can increase the sensitivity of anti PD-L1 therapy in tumors by up regulating the expression of PD-L1 (122). In addition, normalization of tumor blood vessels promotes tumor infiltration of activated T cells after immunotherapy. Recent studies have also found that the combination of drugs can increase CD8^+^ T cell infiltration in HCC by inducing CXCL10 expression (123).

In several preclinical studies, angiogenesis inhibitors improved the efficacy of α-PD-1/PD-L1 in mouse tumor models (123–125). In the IMBrave150 study, atezolizumab combined with bevacizumab was shown to significantly improve the prognosis of patients and to maintain a clinically significant survival benefit after a longer follow-up compared to sorafenib (126).

Following the success, another successful combination was evaluated in ORIENT-32, the first clinical study of combined PD-1 therapy for HCC in the world to reach the primary endpoint. It is a phase 2–3 randomized open-label study that aimed to test the effectiveness of sintilimab plus a biosimilar bevacizumab (IBI305) versus sorafenib as first-line treatment in unresectable HCC in a Chinese population with predominant in HBV infection. The primary endpoints were OS and PFS, both of which were reached (127). The National Medical Products Administration(NMPA) officially approved the combination for the first-line treatment of unresectable or metastatic HCC without prior systematic treatment.

It is worth noting that anti VEGF therapy may promote the recovery of immune response through the above mechanisms, but over pruning the tumor vascular system may aggravate the hypoxia in the tumor microenvironment, thereby increasing immunosuppression (128). Hypoxia can promote the expression of immunosuppressive cytokines and increase the infiltration of immunosuppressive cells to induce immunosuppression (129). Therefore, in order to overcome the immunosuppression caused by VEGF blocking, there may be two directions to consider: 1.TKI and anti PD-1 therapy and targeted hypoxia triple therapy (130). 2. Another method is to carefully titrate VEGF inhibition to inhibit VEGF pathway and angiogenesis while at the same time avoiding over pruning and hypoxia (128). In addition, compared with antiangiogenic agents, the biochemical hybrid and potential off target-toxicity of TKIs have potential limitations in combination therapy (131).For example, in COSMIC-312, 54% of the patients had adverse events of grade 3 or 4 after receiving the combined treatment of cabozantinib plus atezolizumab, and 6 (1%) patients in the combination therapy group had treatment-related grade 5 events, while IMbrave150 did not show such a serious increase AEs (78).

### 6.3 Combination with anti-CTLA-4 antibodies

Cytotoxic T lymphocyte-associated antigen 4, a member of the CD28-B7 superfamily, is located on the surface of activated T cells and acts as an inhibitor of T cell activation. CTLA-4 is a competitive homologue of CD28, with a higher affinity for CD80 and CD86 (B7-1). After binding to the ligand, co-stimulation of T cells is inhibited due to the lack of activation of the second signal (132–134). Moreover, the combination of CTLA-4 and CD80/CD86 counteracts TCR induced downstream signal transduction through PP2A and inhibits PI3K Akt pathway. In addition, CTLA-4 can capture its ligand through a process of trans-endocytosis and degrade it, further hindering costimulatory signals (6).

Although CTLA-4 and PD-1 are both negative regulators of T cells, they both play a different role in the co-inhibitory mechanism of the immune response. CTLA-4 mainly plays a role in lymph nodes at the early stage of T cell immune response, while PD-1 mainly plays a role in peripheral tissues at the late stage of T cell immune response (135). Therefore, blocking PD-1/PDL-1 axis and CTLA-4 axis at the same time plays a nonredundant role in restoring immune activity. In addition, PD-L1 plays an immune stimulating role by inhibiting CTLA-4 axis, while anti PD-L1 reduces the cell surface expression of CD80 on APC, and this effect was negated by co-blockade of CTLA-4 (136).

Ipilimumab is a monoclonal antibody that can block the binding of CD80/CD86 ligands on APCs to CTLA-4 receptors on activated T cells, thus removing immunosuppressive signals and allowing for T-cell priming and clonal expansion. When combined with Nivolumab (PD-1 inhibitor), it can achieve the follow-up anti-tumor function of effector T cells. Moreover, ipilimumab can cause ADCC mediated Tregs cleavage and reduce Tregs infiltration *in vitro*, enhancing the anti-tumor activity of combined drugs (137). In cohort 4 of the CheckMate 040 trial, the safety and efficacy of a combination regimen of nivolumab and ipilimumab were demonstrated in patients treated with sorafenib (138).

Another combination of tremelimumab + durvalumab as second-line trial for advanced HCC was presented at the 2020 ASCO Annual Meeting. The experiment was divided into four regimens of T300+D, T75+D, monotherapy tremelimumab, and monotherapy durvalumab. In the T300+D group, the ORR was 22.7%, mOS was 18.7 months, and DOR was not reached, which was the best among the four groups (139). In the latest data, the T300+D group still had the best risk-return effect (140). Based on these results, a Phase III HIMALAYA study is being conducted to compare the clinical efficacy and safety of the combination of the two drugs versus sorafenib as first-line therapy for advanced HCC (141). The latest results showed that the combination regimen reduced the risk of death by 22% compared to the sorafenib group (P=0.0035). The median overall survival (OS) in both groups was 13.8 months versus 16.4 months, meeting the primary endpoint; PFS was 3.78 months versus 4.07 months, ORR was 20.1% VS 5.1%; DOR was 22.34 months vs 18.43 months; DCR was 60.1% vs 60.7%, and the incidence of treatment-related adverse events of grade 3 or 4 was 25.8% vs 36.9%. Based on these results, AstraZeneca announced an application to the US Food and Drug Administration for approval of the STRIDE regimen that applies durvalumab plus tremelimumab as a first-line treatment for unresectable HCC (142).

A randomized, open and controlled multicenter phase III clinical study (NCT04720716) aims to test the effectiveness of the combination of IBI310 (anti-CTLA-4 monoclonal antibody) combined with the PD-1 inhibitor sintilimab for the first-line treatment of advanced HCC is also ongoing.

### 6.4 Combination with radiotherapy

In the early years, the results of radiotherapy (RT) for HCC were not satisfactory because radiotherapy may lead to radiation-induced liver disease (RILD) and the relationship between radiation dose and tumor could not be determined (143, 144). However, as technology has improved, new techniques such as stereotactic body radiation therapy (SBRT) and radioembolization (RE) have been able to deliver high doses of radiation to the tumor while limiting damage to surrounding healthy tissue.

Local radiotherapy can induce immunogenic cell death and produce DAMPs and cytokines and/or chemokines in the TME to directly or indirectly promote immune cell recruitment through antitumor and protumor effects (145); it can increase the expression of major histocompatibility complex I (MHCI) in tumor cells (146). Radiation can also repolarize macrophages into tumor suppressing M1 subtypes and activate natural killer cells (147). SABR delivers 3 to 5 segments of highly conform-focused high-dose radiation to the target through small edge and daily imaging (usually cone-beam CT) (146), while RE is an internal radiation technique that utilizes β-emission, where radiation-labeled microembolic particles (e.g., Y-90) are introduced directly into HCC through the liver artery with limited tissue penetration (148).

These techniques have made radiotherapy more and more widely used in the treatment of HCC. However, radiation can also promote massive lymphatic depletion (143), reduce antigen expression, and lead to elevation of circulating immunosuppressive cells (149), and the use of -PD-1/PD-L1 can reduce the immunosuppressive response. In arterial chemoembolization (TACE), for example, ischemic injury induced by arterial occlusion induces hypoxia-inducible factor (HIF-1α), a known regulator of PD-L1 expression, which can be blocked by α-PD-1/PD-L1. Furthermore, TACE can act on the tumor microenvironment by decreasing the percentage of T-Reg and increasing the ratio of CD4+/CD8+, suggesting that TACE may interact with the antitumor activity of ICI (150). Recent research results have also shown that the addition of anti-PD-1 therapy to radiation can enhance the abscopal effect of HCC (151).

Clinical and preclinical studies have shown that RT can synergize with α-PD-1/PD-L1 in a variety of ways. First, radiotherapy promotes T cell infiltration, increases the number of TILs, and expands the TCR pool in the TME. Second, radiotherapy upregulates the expression of PD-L1 in tumor cells, which can be blocked by α-PD-1/PD-L1. Third, radiation therapy increases MHC-I in tumor cells and removes resistance to α-PD-1/PD-L1 (6). Some ongoing clinical trials are shown in **Table 4**.

**Table 4:** Ongoing and upcoming clinical trials investigating combination therapies in HCC.

Trial Name, Number	Phase	a-PD-1/PD-L1	Combined Therapy	Estimated Sample Size	Status	Primary Outcome Measures
NCT04829383	II	Atezolizumab	Bevacizumab (VEGF- targeting)	50	Recruiting	Frequency and severity of toxicities
NCT04770896	II	Atezolizumab	Lenvatinib (TKI)	554	Recruiting	OS
NCT04826406	III	Camrelizumab	Apatinib (TKI)	40	Recruiting	ORR
NCT04443309	I/II	Camrelizumab	Lenvatinib (TKI)	53	Recruiting	ORR
NCT05048017	II	Camrelizumab	Regorafenib (TKI)	20	Recruiting	PFS
NCT04806243	II	Camrelizumab	Regorafenib (TKI)	69	Recruiting	OS
NCT04310709	II	Nivolumab	Regorafenib (TKI)	42	Recruiting	ORR
NCT04170556	I/II	Nivolumab	Regorafenib (TKI)	78	Recruiting	Rate of AE, related AEs, death
CheckMate 9DW NCT04039607	III	Nivolumab	Ipilimumab (CTLA-4-targeting)	728	Recruiting	OS
NCT05199285	II	Nivolumab	Ipilimumab (CTLA-4-targeting)	40	Not yet recruiting	ORR
NCT05096715	1B	Atezolizumab	Bevacizumab (VEGF targeting) + stereotactic beam radiation therapy	20	Not yet recruiting	Dose limiting toxicity Rate
NCT03482102	II	Durvalumab	Tremelimumab (CTLA-4-targeting) + radiation therapy	70	Recruiting	BOR
NCT05488522	I	Atezolizumab	Bevacizumab (VEGF targeting) + stereotactic beam radiation therapy			

OS, Overall Survival; BOR, Best Overall Response.

### 6.5 Other combinations

#### 6.5.1 Combination with targeting HIF-1α

A major limitation of the anti-PD-1/anti-PD-L1 monoclonal antibodies is that they cannot distinguish PD-1 – PD-L1 interactions between TME and normal tissues. It may be possible to differentiate and improve the drug efficacy according to the different molecular mechanisms of PD-L1 expression in normal tissues and cancers. For example, hypoxia is one of the main indicators to distinguish solid tumors from normal tissues. Hypoxia can induce PD-L1 expression in malignant and immunomodulatory cells through HIF-1 α (152). Recent experimental results show that the combined application of pharmaceutical or genetic targeting of HIF-1α can inhibit the expression of PD-L1 in TME. Besides it meanwhile induces PD-L1 in normal tissues through increasing the IFN – γ produced by T cells, protecting normal tissues from immune damage (152). In the HCC mouse model, HIF inhibitor 32-134D combined with anti PD1 treatment can increase the tumor eradication rate from 25% to 67%. Researchers speculate that 32-134D changes the expression of a large number of genes, leading to significant changes in the tumor immune microenvironmen, and the percentage of CD8+T cells and natural killer cells, thus significantly enhancing anti PD1 therapy (153). Therefore, targeting HIF-1α combined with anti PD1 therapy may be a breakthrough treatment for HCC.

#### 6.5.2 Combination with IFN α

Type I interferon (IFN) (154) has been proved to be able to directly and indirectly inhibit tumor growth by acting on tumor and immune cells, and has synergistic effect against tumor immunity. IFN- α is a kind of cytokine belonging to type I interferon family. Depending on its anti-tumor activity, it has become a potential drug that can be combined with new therapeutic strategies to treat cancer (155, 156). Study shows that The combination of PD-1 blockade with IFN α significantly improved the effect of PD-1 antibody monotherapy, prolonged the survival period of mice, enhanced the secretion and activation of T cells in liver cancer mouse models, restored or even enhanced the cytotoxic effect of CD8+T cells, and had a synergistic anti-tumor effect (157). A recent study revealed that the mechanism may due to that the combination can damage the glycolysis and glucose uptake of HCC cells, reshape and form a glucose rich tumor microenvironment (TME), which can enhance the killing function of tumor infiltrating cytotoxic T lymphocytes. In addition, the high glucose environment induced the protein level of CD27 and other molecules in CD8+T cells to increase, which restored the anti-tumor effect of CD8+T cells by increasing CD27 transcription (158).

## 7 Conclusions

The unique immunobiological characteristics of the liver not only prevent it from being immune damaged, but also create an environment for tumor immune tolerance, in which the PD-1/PD-L1 pathway undoubtedly plays a huge role. In this article, we review the mechanism of PD-1/PD-L1 blockade and its efficacy and safety in clinical trials. According to current clinical results, the PD-1/PD-L1 antibody may be more suitable for combination therapy. Currently, the most common programs include combination with antivascular drugs, dual immunotherapy, and combination with radiation therapy and chemotherapy. However, the prediction of tumor biomarkers, the efficacy of drug therapy, and the study of adverse drug reactions still need a lot of basic and exploratory research. The development of PD-1/PD-L1 blocking therapy will undoubtedly be a great opportunity and challenge. We hope that future research can minimize drug resistance, reduce the occurrence of immune-related adverse events, and improve the efficacy of immunotherapy.

## Author contributions

QL wrote the draft of the manuscript and contributed to the completion of figures and tables. JH contributed to the design and approval of the submitted version. All authors contributed to the article and approved the submitted version.
